# How AI-Generated Content Shapes User Trust: The Roles of Cognitive Processing, Perceived Risk, and Transparency Labels

**DOI:** 10.3390/bs16060957

**Published:** 2026-06-10

**Authors:** Junting Liu, Liangdong Lu

**Affiliations:** Business School, Hohai University, Nanjing 211100, China

**Keywords:** artificial intelligence, AI-generated content, cognitive processing, trust, perceived risk, adoption intention, sharing intention, transparency labels

## Abstract

Generative artificial intelligence (AI) is increasingly used to produce multimedia content, raising important questions about how users evaluate and respond to such content. While prior research has examined AI transparency and technology adoption, the cognitive mechanisms linking external cues to user trust and behavioral outcomes remain insufficiently understood. Grounded in the heuristic–systematic model (HSM), this study investigates how transparency labels and presentation quality influence cognitive processing, perceived authenticity, trust, and perceived risk in AI-generated video content. A 2 × 2 between-subjects experiment was conducted with 617 participants. The results show that AI transparency labels reduce perceived authenticity but do not directly affect trust or adoption intention. Higher presentation quality reduces reliance on heuristic processing. Perceived authenticity is associated with lower cognitive load and greater systematic processing, systematic processing is positively associated with trust, whereas heuristic processing is negatively associated with trust. Trust is positively associated with both adoption intention and sharing intention, whereas perceived risk is negatively associated with these outcomes. These findings highlight the central roles of cognitive processing and trust–risk dynamics in shaping user responses to AI-generated content and provide implications for transparency design and trust development in AI-mediated environments.

## 1. Introduction

The rapid development of generative artificial intelligence is profoundly transforming digital content creation and multimedia information environments. As the capabilities of large language models continue to improve, AI-generated content has expanded from text to images, audio, and video. Due to its low production costs, high dissemination efficiency, and scalability, AI-generated video content is becoming a prominent format on digital platforms (Huang et al., 2025; Park, 2025). Unlike previous systems—such as intelligent recommendation or automated backend tools—which were often embedded in the background, generative AI content now enters users’ direct viewing processes in a more explicit and visible manner. Consequently, how users understand, evaluate, and respond to such content has become a critical issue in human–computer interaction and digital media research. Research on generative AI tools such as ChatGPT also indicates that while the technology is diffusing rapidly, user willingness to adopt it, the foundations of trust, and the boundaries of its application remain in flux (Y. Li et al., 2025; Zhang et al., 2025).

However, the proliferation of AI-generated video content does not merely signify improved efficiency; it is also accompanied by concerns regarding authenticity, accountability, the spread of misinformation, and ethical standards. In particular, as platforms and regulators increasingly emphasize that AI-generated content should be labeled, transparency labels have become a key tool for content governance. A seemingly straightforward inference is that such disclosure should enhance users’ trust in platforms or content creators, as it signals honesty and responsibility. Yet existing research has not provided a consistent answer. A growing body of evidence suggests that AI-related information may enhance perceptions of transparency while simultaneously triggering greater vigilance regarding content reliability, perceived authenticity, and potential risks (F. Li & Yang, 2024; Schilke & Reimann, 2025). In other words, transparent labeling does not necessarily translate into user acceptance; instead, it may activate a more cautious and risk-aware evaluation mode.

Existing research has addressed related issues from several distinct perspectives, including technology acceptance, AI trust, and content evaluation. One line of research emphasizes that various cognitive, social, and situational factors influence intentions to adopt new technologies, which in turn affect actual usage behavior; another highlights that trust serves as a pivotal mediating variable in human–AI interactions, directly influencing collaborative and usage intentions while also mediating between antecedent variables and behavioral intentions (Y. Li et al., 2025; Zhang et al., 2025). Meanwhile, research on AI disclosure, content sources, and information credibility has shown that external labeling cues can significantly influence users’ initial judgments of content (F. Li & Yang, 2024; H. Chen et al., 2025). However, most of these studies focused on general AI systems, chatbots, or short-form video and information content, and discussions of the specific context of AI-generated video content—which combines informational and media characteristics—remain limited. More importantly, existing research has rarely attempted to explain simultaneously how external cues shape users’ front-end information processing and how such processing further influences acceptance and recommendation through trust and perceived risk.

These gaps motivate the present study. Unlike general AI tools, AI-generated video content is a content-rich audiovisual format. Users face not merely a decision of whether to use an AI tool, but rather a continuous judgment process involving whether to trust a given piece of content, whether to continue engaging with such content, and whether to recommend it to others. In other words, AI-generated video content is not simply a technical artifact, but a complex object that encompasses source information, presentation characteristics, and dissemination consequences. Therefore, relying solely on a technology acceptance framework is insufficient; research must further explain how users process information when encountering AI-generated video content and how this processing translates into deeper assessments of trust and perceived risk.

On this basis, the present study introduces the heuristic-systematic model (HSM) as the core framework for explaining users’ front-end cognitive processing. The HSM posits that individuals allocate cognitive resources to varying degrees between systematic processing and heuristic processing: the former relies on in-depth analysis and evidence integration, while the latter depends more on surface cues, intuitive impressions, and simplified judgments (Chaiken, 1980; S. Chen & Chaiken, 1999). In the context of AI-generated video content, perceived authenticity and cognitive load are expected to influence which processing mode users tend to adopt, and these different processing modes, in turn, further shape their trust in the content and its source. At the same time, trust alone is insufficient to fully explain user behavior, as generative AI content also elicits uncertainty and perceived risk. For this reason, the present study further incorporates perceived risk into the model, together with trust constituting the proximal mechanisms for explaining adoption intention and sharing intention.

Building on this foundation, the study further introduces a signaling perspective to understand the roles of AI labels and video quality. Signaling theory posits that under conditions of information asymmetry, recipients often rely on observable cues to infer qualities or attributes that cannot be directly observed; the more salient the external signals provided by the sender, the more likely they are to influence the recipient’s initial judgment and subsequent responses. Classic signaling theory centers on how observable signals help recipients infer hidden attributes, and subsequent reviews have emphasized its applicability to situations in which one party lacks information and the other makes judgments based on observable cues (Spence, 1973; Connelly et al., 2011). In the context of AI-generated video content, the AI label can be viewed as a source signal indicating how the content was produced, while video quality can be viewed as a presentation signal influencing users’ initial inferences about the content’s professionalism, rigor, and processing value. Accordingly, this study integrates “AI label—video quality—cognitive processing—trust/risk—behavioral intention” into a more comprehensive explanatory chain.

To examine these issues, this study employs a 2 (AI label: present/absent) × 2 (video quality: high/low) between-subjects experiment, using AI-generated video content as stimulus materials, to test whether these two types of external cues influence users’ perceived authenticity, cognitive processing styles, trust, perceived risk, and subsequent behavioral intentions. Adoption intention serves as the focal outcome variable. Sharing intention is examined as a parallel outcome variable; user literacy is examined as a boundary-condition moderator in Section 4.5, to assess the broader applicability and boundary conditions of the core findings, given that willingness to recommend content to others may reflect distinct mechanisms related to social responsibility and dissemination judgment (F. Li & Yang, 2024; Y. Li et al., 2025).

Based on the above analysis, this study seeks to answer the following core questions: In the context of AI-generated video content, how do AI labels and video quality, as external cues, influence users’ information processing? Also, how does this influence further affect adoption intention through trust and perceived risk?

This study makes three main contributions. First, by focusing on AI-generated video content—a form of technology-mediated content—this study extends research on generative AI acceptance from general tool usage to content evaluation and acceptance. Second, this study introduces the heuristic–systematic model to explain users’ front-end cognitive processing and by integrating a signaling perspective, defines AI labels as the primary source signal and video quality as a supplementary presentation signal influencing initial judgments, thereby constructing an integrated “external cues—cognitive processing—trust/risk—behavioral intention” framework. Third, the findings indicate that AI labels do not automatically yield a so-called “transparency dividend”; rather, they primarily alter perceived authenticity. Users’ ultimate acceptance appears better explained by two proximal pathways: enhanced trust and reduced perceived risk. These findings offer practical implications for digital platforms regarding the design of AI labels, the optimization of content presentation, and the implementation of trust management strategies.

## 2. Literature Review and Research Hypotheses

### 2.1. External Cues and Initial Judgments in AI-Generated Video Content

A prominent feature of generative artificial intelligence content is that its source information itself can serve as a crucial cue for users when judging content quality and reliability. In digital content dissemination scenarios, users often cannot directly verify the content generation process or information sources; therefore, they rely on external labels, platform descriptions, or content presentation characteristics to form initial judgments. For AI-generated video content, these external cues primarily manifest in two categories: source cues, namely whether the content is explicitly labeled as “AI-generated”; and presentation cues, which refer to the quality of the video in terms of visuals, audio, and overall production (H. Chen et al., 2025; Pfänder & Altay, 2025). Under these conditions, external cues are important not merely because they are easily observable, but because they serve a signaling function under conditions of information asymmetry: users rely on these observable cues to infer the content’s production method, professionalism, and potential reliability. Classic signaling theory and subsequent reviews emphasize that recipients often rely on overt signals to infer qualities and attributes that cannot be directly observed (Spence, 1973; Connelly et al., 2011).

In this context, AI labels serve not only as technical descriptions but may also be perceived by users as key signals influencing their perceived authenticity and credibility judgments. When users learn that content is AI-generated, their assessment of the content’s authenticity may no longer rely solely on the information presented in the video itself. Instead, they may associate it with potential issues such as automated generation, lack of human review, and possible information distortion, thereby reducing their perceived authenticity (F. Li & Yang, 2024; Schilke & Reimann, 2025). At the same time, video quality, as another external cue, may also influence users’ perceptions of the content’s professionalism, clarity, and comprehensibility, thereby altering their subsequent judgment pathways. It should be noted that the video quality discussed in this study is primarily limited to audiovisual presentation quality, rather than broader dimensions such as content quality, structural quality, or presenter performance quality. In this study, video quality is not treated as a general proxy for overall content quality but is defined as a presentation cue that users can most readily perceive during the initial viewing stage. For this reason, this study primarily treats it as an external presentation signal and examines whether and how it alters users’ initial judgments and processing pathways regarding AI-generated video content. For video content, higher presentation quality not only implies a better sensory experience, but may also be interpreted by users as a signal of greater professionalism and investment, thereby influencing their subsequent processing (H. Chen et al., 2025).

Based on the above analysis, this study defines AI labels as the primary source signal and video quality as a supplementary presentation signal in the context of AI-generated video content. Specifically, AI labels primarily function as source signals influencing users’ perceived authenticity, while video quality primarily acts as a presentation signal affecting the starting point of users’ information processing. Consequently, the following hypothesis is proposed:

**H1.**  *AI labels significantly reduce users’ perceived authenticity of AI-generated video content.*

### 2.2. Perceived Authenticity, Cognitive Load, and Information Processing Modes

The heuristic–systematic model posits that individuals do not always engage in thorough analysis when processing information. Instead, under the combined influence of cognitive resources, task motivation, and external cues, they allocate attention between systematic processing and heuristic processing. Systematic processing relies on in-depth analysis, evidence integration, and logical judgment, whereas heuristic processing relies more on surface cues, intuitive impressions, and simplified judgments (Chaiken, 1980; S. Chen & Chaiken, 1999). For video content—a content format characterized by high information density and a certain comprehension threshold—users’ willingness to invest cognitive resources in in-depth processing is often jointly influenced by their perceived authenticity of the content and their cognitive load.

First, perceived authenticity is a crucial prerequisite for subsequent processing. If users perceive video content as authentic and reliable, they are more likely to invest greater attention in evaluating the content’s logic, argument structure, and explanatory processes in depth, thereby tending toward systematic processing. Conversely, if users doubt the content’s authenticity, they are more likely to reduce in-depth analysis and instead rely on surface cues and quick impressions to form judgments (H. Chen et al., 2025). In other words, perceived authenticity influences not only content evaluation itself, but also whether users are willing to invest cognitive resources in that content.

Second, cognitive load is another key variable influencing information processing mode. Systematic processing requires more cognitive resources, whereas heuristic processing is a relatively resource-efficient mode of judgment (Petty & Cacioppo, 1986). When individuals perceive information as complex, the cost of understanding as high, or their cognitive resources as limited, they are more likely to reduce in-depth analysis and instead rely on simple cues, intuitive impressions, or superficial features to form judgments. This is particularly relevant for AI-generated video content. Unlike general entertainment short videos, this type of video content often requires relatively high cognitive engagement, such as logical integration and conceptual processing. If users perceive the video content as difficult to understand, poorly organized, or cognitively taxing, their willingness to engage in systematic processing is likely to diminish, while their tendency toward heuristic processing is likely to increase (Kendeou & Johnson, 2024; Peng et al., 2024).

Based on this, the present study posits that perceived authenticity and cognitive load are two core antecedents influencing users’ processing of AI-generated video content. Consequently, the following hypotheses are proposed:

**H2.**  *Higher perceived authenticity is associated with lower perceived cognitive load.*

**H3.**  *Higher perceived authenticity is associated with greater engagement in systematic processing.*

**H4.**  *Higher cognitive load is associated with less engagement in systematic processing.*

**H5.**  *Higher cognitive load is associated with greater engagement in heuristic processing.*

### 2.3. Information Processing Modes, Perceived Risk, and Trust Formation

In AI contexts, trust is typically viewed as a key psychological mechanism linking cognitive judgments to behavioral intentions. Existing research indicates that trust not only directly influences individuals’ willingness to use AI systems, their willingness to collaborate, and their level of acceptance, but also mediates the relationship between antecedent variables and behavioral outcomes (Y. Li et al., 2025; Schilke & Reimann, 2025). For AI-generated video content, whether users trust the content itself and its creators is often not directly determined by a single external cue but is gradually formed during the cognitive processing process.

From the perspective of the heuristic–systematic model, systematic processing implies that users carefully analyze video information, integrate evidence, and evaluate its plausibility; consequently, they are more likely to develop a relatively stable sense of trust based on thorough judgment. In contrast, heuristic processing relies more on superficial features and quick impressions; while this helps reduce cognitive effort, it may also result in trust judgments that lack a solid informational foundation. In the context of AI-generated video content, if users form trust primarily on the basis of superficial cues, their trust is more susceptible to uncertainty and preconceptions; whereas when users engage in systematic processing to thoroughly evaluate the content’s logic and information quality, trust is more likely to be grounded in the content itself (Chaiken, 1980; S. Chen & Chaiken, 1999).

Furthermore, AI-generated video content involves not only the establishment of trust but also concerns about potential misinformation and uncertainty. When users perceive video content as highly authentic and reliable, their concerns about the negative consequences that the content might entail tend to decrease accordingly. At the same time, in the context of generative AI content, perceived risk and trust are not mutually exclusive but exhibit a trade-off relationship: the higher the perceived risk, the more difficult it is for users to form stable trust; conversely, the lower the perceived risk, the easier it is to establish trust (Featherman & Pavlou, 2003; Y. Li et al., 2025).

Based on the above analysis, the following hypotheses are proposed:

**H6.**  *Systematic processing is positively associated with user trust.*

**H7.**  *Heuristic processing is negatively associated with user trust.*

**H8.**  *Perceived authenticity is negatively associated with perceived risk.*

**H9.**  *Perceived risk is negatively associated with user trust.*

Specifically, perceived risk in this context comprises three content-related dimensions: information accuracy risk (concerns about factual errors or inaccuracies), learning consequence risk (concerns that over-reliance on AI may negatively affect learning outcomes), and general negative consequence risk (concerns about broader adverse effects of adopting such content). Privacy and data security risks were outside the scope of this study, as the experimental context involves passive video viewing rather than interactive AI use or personal data submission.

### 2.4. Theoretical Relationships Among Trust, Perceived Risk, and Adoption Intention

Users’ ultimate response to AI-generated video content is primarily reflected in their willingness to continue engaging with such content. Adoption intention reflects an individual’s overall propensity to adopt this type of technology and content format, and thus serves as the focal outcome variable for measuring user responses to AI-generated video content. Existing AI research generally indicates that trust is a key proximal variable in fostering behavioral intentions. The more individuals believe that AI systems or AI content are reliable, beneficial, and trustworthy, the more likely they are to continue using or accepting such content. Recent studies of AI-mediated interaction also show that users’ intention to adopt AI systems is influenced by various proximal factors, among which trust-related judgments constitute a key component (Guo & Erdenebold, 2025).

At the same time, generative AI content is often accompanied by perceived risks, such as potential inaccuracies, misleading information, unclear accountability, or inconsistent response outcomes. Such perceived risks may inhibit users’ further acceptance of the content. In other words, trust and perceived risk are not merely ancillary variables but rather two proximal pathways influencing adoption intention for AI-generated video content: the former represents a positive facilitation mechanism, while the latter represents a negative inhibition mechanism (Featherman & Pavlou, 2003; Kendeou & Johnson, 2024). This argument aligns with TAM-based mobile banking research, which demonstrates that security and privacy concerns can inhibit adoption decisions regardless of perceived ease of use (Bakar et al., 2017). It also parallels broader e-service adoption research identifying perceived risk as a fundamental barrier to technology acceptance (Featherman & Pavlou, 2003).

Based on this, the following hypotheses are proposed:

**H10.**  *Trust is positively associated with adoption intention.*

**H11.**  *Perceived risk is negatively associated with adoption intention.*

Furthermore, given that sharing intention is an extended outcome variable pertaining to the content dissemination level, this study further examines the effects of trust and perceived risk on sharing intention in Section 4.5, proposing the following hypotheses:

**H12.**  *Trust is positively associated with sharing intention.*

**Figure 1:**
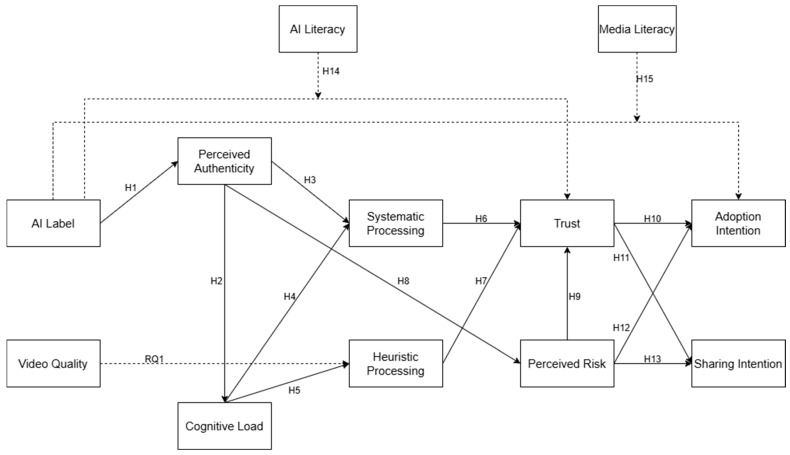
Conceptual model and research hypotheses. Solid arrows represent the hypothesized structural pathways (H1–H13), including the core adoption-intention pathways and the extended sharing-intention pathways. The dashed arrow from Video Quality to Heuristic Processing represents RQ1, and the dashed arrows from AI Literacy and Media Literacy indicate the literacy-related moderation paths (H14–H15) tested as boundary conditions.

**H13.**  *Perceived risk is negatively associated with sharing intention. H12 and H13 are included in Figure 1 alongside the core model pathways and are reported in Section 4.5.*

### 2.5. The Moderating Role of User Literacy

In addition to external cues and cognitive processing, individual user differences may also influence responses to AI-generated video content. This study focused on two types of competence variables: AI literacy and media literacy. AI literacy reflects an individual’s level of understanding regarding the principles, application boundaries, and potential limitations of artificial intelligence technology; media literacy reflects an individual’s ability to identify, analyze, and evaluate media content, which serves as a critical cognitive resource for users when navigating technology-mediated information environments (Ashley et al., 2013; Livingstone, 2004). For the same “AI-generated” label, users with different literacy levels may arrive at different interpretations: users with high AI literacy may be better able to distinguish between “AI-generated” and “necessarily untrustworthy”, and thus may not necessarily experience a significant decline in trust due to the label; users with high media literacy, on the other hand, may attend more closely to content evaluation criteria and source information, thereby exhibiting acceptance tendencies that differ from those of the general user population (Ng et al., 2021; Long & Magerko, 2020).

Recent research indicates that AI literacy influences individuals’ understanding of, reliance on, and trust in AI systems, while media literacy helps enhance users’ ability to discern misinformation, manipulative content, and digital source cues (Zhang et al., 2025; Peng et al., 2024; Anstead et al., 2025). Therefore, this study treats AI literacy and media literacy as potential boundary conditions for the AI label effect and further examines their moderating roles in Section 4.5, proposing the following hypotheses:

**H14.**  *AI literacy moderates the association between AI labels and user trust.*

**H15.**  *Media literacy moderates the association between AI labels and users’ adoption intention.*

### 2.6. Conceptual Definitions and Measurement Boundaries

To clarify the theoretical content and sources of the core constructs in this study, Table 1 summarizes the definitions of the main constructs and their core literature sources. This table primarily serves to illustrate the theoretical foundations and conceptual boundaries of each construct, rather than to present specific scale items. For specific items, item numbers, and measurement sources, see Appendix A Table A1.

It is necessary to clarify the conceptual boundary between perceived authenticity and trust in this context. Perceived authenticity refers to users’ judgment of whether content is genuine, accurate, and unmanipulated—a content-level evaluation that precedes trust formation (F. Li & Yang, 2024; Schilke & Reimann, 2025). Trust, in contrast, reflects a broader relational assessment of the reliability and dependability of the content source and its generation mechanisms, and is shaped by prior content evaluations as well as accumulated experience (McKnight & Chervany, 2001; Y. Li et al., 2025). In other words, authenticity judgments function as an antecedent input to trust rather than as a substitute for it.

### 2.7. Research Model

Based on the above analysis, this study constructed an integrated model of “external cues—cognitive processing—trust/risk—behavioral intention”. In this model, AI labels and video quality constitute external cues; from a signaling perspective, the former primarily serves as a source signal, while the latter primarily serves as a presentation signal, jointly influencing users’ initial judgments of AI-generated video content. Furthermore, perceived authenticity and cognitive load influence systematic and heuristic processing; systematic and heuristic processing in turn affect trust; perceived authenticity influences perceived risk, which in turn inhibits trust; and ultimately, trust and perceived risk jointly influence adoption intention.

It should be noted that given the lack of consensus in existing research regarding the direction of video quality’s effects in AI content evaluation, and given that video quality is incorporated into the experimental design primarily as an external presentation signal, this study does not presuppose strong directional hypotheses for all of its effect pathways. Instead, beyond the hypothesis framework, the following exploratory research question is proposed:

RQ1: As a presentation signal, does video quality influence users’ initial cognitive processing when encountering AI-generated video content—particularly their tendency toward heuristic processing—and if so, how?

Furthermore, this study examines the relationships involving sharing intention and user literacy to assist in evaluating the generalizability and boundary conditions of the core model’s conclusions. Sharing intention pathways (H12, H13) are included in Figure 1 as parallel outcome pathways. The literacy moderation paths (H14, H15) are represented by dashed arrows as boundary conditions. This approach preserves the integrity of the experimental design while avoiding overly strong assumptions about the effects of video quality when theoretical evidence remains insufficient. Additionally, this study does not equate “video quality” with general content quality but rather limits it to an audiovisual presentation quality that users can immediately perceive. The core research model is shown in Figure 1.

## 3. Methods

### 3.1. Research Design and Experimental Materials

This study employed a 2 (AI label: present/absent) × 2 (video quality: high/low) between-subjects experimental design, using AI-generated video content as stimulus materials, to examine the effects of two types of external cues—AI labels and video quality—on users’ perceived authenticity, cognitive processing styles, trust, perceived risk, and behavioral intentions (Charness et al., 2012). Building upon the integrated framework of “external cues—cognitive processing—trust/risk—behavioral intention” established in the preceding sections, the experiment included four conditions: low quality + no label, low quality + AI label, high quality + no label, and high quality + AI label.

The stimulus material was a short explanatory video introducing the “Pomodoro Technique”. The research team first established a unified content topic and script, then used OpenAI’s Sora to generate the base video footage. To ensure consistency across the four experimental conditions at the content level, the research team subsequently standardized the generated output, including proofreading the narration text, standardizing the sequence of scenes, and controlling subtitles and duration. This ensured that videos across all conditions remained consistent in core content, explanatory logic, narration text, and overall duration. This process resulted in a single, content-consistent original video version, upon which AI labels and video quality manipulations were further applied. Notably, the low-quality version was not regenerated but was derived from the same original video through downsampling and compression, ensuring that between-group differences stemmed primarily from experimental manipulation rather than from the content itself. Based on these procedures, this study employed a “same-source video with condition-specific manipulation” approach to construct the four experimental conditions.

Regarding video quality manipulation, the high-quality condition featured clearer and smoother audiovisual presentation; under the low-quality condition, the same original video underwent downsampling and compression, resulting in noticeably lower resolution, image clarity, and visual detail compared to the high-quality version, thereby simulating common viewing experiences across different devices, bandwidths, or service tiers. It should be noted that “video quality”, as used in this study, primarily refers to audiovisual presentation quality and does not involve differences in the underlying content itself.

Regarding AI label manipulation, under the “AI label present” condition, a prominent “AI-generated” label appeared in the upper-left corner of the video; under the “AI label absent” condition, this label was not displayed. After random assignment, participants first watched the video stimulus corresponding to their experimental condition and then completed a questionnaire measuring perceived authenticity, cognitive load, systematic processing, heuristic processing, trust, perceived risk, adoption intention, sharing intention, AI literacy, and media literacy.

### 3.2. Sample and Procedure

This study recruited participants through the Credamo survey platform, collecting a total of 640 questionnaires. Specifically, participants were restricted to currently enrolled students aged 18–25 years, representing the primary audience for AI-generated video content on digital platforms. The target sample size was set to approximately 160 participants per experimental condition, consistent with conventions in comparable 2 × 2 between-subjects experimental research. Post hoc sensitivity analysis confirmed that the final sample size of 617 provided a statistical power exceeding 0.99 to detect medium-sized effects (f = 0.25) in an ANOVA framework (Faul et al., 2007; Lakens, 2022). To ensure data quality, attention check items were included in the questionnaire, and data were cleaned according to predefined criteria, primarily excluding participants who failed the attention checks or who exhibited clear inconsistencies in experimental manipulation recognition. After cleaning, the final sample used for model estimation and hypothesis testing comprised 617 participants, yielding a valid response rate of 96.4%.

To describe the basic characteristics of the sample, this study further compiled demographic information including respondents’ gender, age, educational attainment, frequency of watching video content, and frequency of generative AI use. The results are presented in Table 2. Randomization checks indicated that the distributions of gender and educational attainment across the four experimental groups did not differ significantly. Specifically, the chi-square test for gender yielded χ^2^(3) = 0.919, *p* = 0.821; the chi-square test for educational attainment yielded χ^2^(12) = 7.946, *p* = 0.789. Age differences across groups were also non-significant, with the one-way ANOVA yielding F(3, 613) = 0.842, *p* = 0.472. Furthermore, the distributions of video content viewing frequency and generative AI usage frequency across the four experimental groups were also non-significant, with χ^2^(12) = 3.632, *p* = 0.989 and χ^2^(9) = 6.702, *p* = 0.668, respectively. These results indicate that the experimental groups achieved adequate randomization, with no significant demographic differences between groups. Detailed randomization check results are presented in Appendix A Table A2.

This study was conducted in accordance with the Declaration of Helsinki. Formal ethics committee review and approval were not obtained for this study. In accordance with the institutional guidelines for minimal-risk anonymous survey research at Hohai University, the exemption status was determined by the researchers, as the study involved a low-risk, anonymous online questionnaire experiment with adult participants. Specifically, the study involved adult participants aged 18–25 years, and the procedures were limited to exposure to standardized video stimuli and the completion of self-report questionnaire items. The researchers collected no personally identifiable information, requested no sensitive personal data, involved no clinical intervention or deception, and posed no foreseeable physical, psychological, or social risk to participants. The survey was administered through the Credamo online survey platform, and the dataset available to the researchers contained only anonymous responses. Therefore, no formal institutional approval number is applicable. All participants provided informed consent by confirming the online consent statement before participation and were free to withdraw at any time without consequence. All data were used solely for academic research purposes.

Regarding the distribution across experimental groups, the sample sizes for the four conditions were as follows: 154 in the low-quality + no-label group, 154 in the low-quality + AI label group, 154 in the high-quality + no-label group, and 155 in the high-quality + AI label group. The overall allocation was relatively balanced, providing a foundation for subsequent between-group comparisons and interaction effect tests. Table 3 presents the sample distribution across the four experimental conditions.

The experimental procedure was as follows. First, participants accessed the online experimental system via a link and were randomly assigned to one of the four experimental conditions; second, participants watched the AI-generated video content corresponding to their experimental condition; third, participants completed a questionnaire evaluating perceived authenticity, cognitive load, systematic processing, heuristic processing, trust, perceived risk, adoption intention, sharing intention, and individual literacy variables; finally, the final analysis sample was formed based on data cleaning rules, and subsequent statistical analyses were conducted.

### 3.3. Measures

This study measured the following constructs: perceived authenticity, cognitive load, systematic processing, heuristic processing, trust, perceived risk, adoption intention, sharing intention, AI literacy, and media literacy. Except for the experimental manipulation variables, all items were measured using a seven-point Likert scale, ranging from 1 (strongly disagree) to 7 (strongly agree), with higher scores indicating stronger perceptions on the corresponding dimension.

The measurement items for each construct were primarily adapted from established, well-validated scales and were moderately revised to fit the AI-generated video content context. Systematic processing and heuristic processing were primarily based on classic HSM research (Chaiken, 1980; S. Chen & Chaiken, 1999); perceived authenticity, trust, and sharing intention were primarily drawn from research on AIGC labels and information credibility (F. Li & Yang, 2024; Y. Li et al., 2025); adoption intention primarily drew on the behavioral intention construct from UTAUT (Venkatesh et al., 2003); perceived risk primarily drew on classic risk perception scales, with revisions tailored to the generative AI content context (Featherman & Pavlou, 2003); AI literacy and media literacy drew on relevant established scales (Ng et al., 2021; Long & Magerko, 2020; Peng et al., 2024), while the supplementary manipulation check for perceived video quality was adapted from Xu and Sundar (2016). Specific items, item numbers, and primary literature sources are listed in Appendix A Table A1.

Convergent validity was assessed using Cronbach’s α, composite reliability (CR), and average variance extracted (AVE); discriminant validity was examined using the Fornell–Larcker criterion and the HTMT ratio (Fornell & Larcker, 1981; Henseler et al., 2015). Confirmatory factor analysis (CFA) and exploratory factor analysis (EFA) were also conducted to examine the stability of the measurement model structure. The detailed measurement model results are reported in Section 4.1.

### 3.4. Data Analysis

The data analysis in this study proceeded through four stages. All primary analyses were conducted in Python 3.12 using the following packages: statsmodels (v0.14) for OLS regression and moderation analyses, factor_analyzer (v0.5) for EFA, and semopy (v2.3) for CFA. Bootstrap mediation used percentile bootstrap with 3000 resamples implemented via numpy (v1.26) and statsmodels.

First, measurement model evaluation was conducted. After data cleaning, the internal consistency, convergent validity, and discriminant validity of each construct were examined. The formal results are reported based on the refined item sets (Auth = {Auth1, Auth2, Auth4}; Trust = {Trust2, Trust3}), from which two double-barreled or referent-inconsistent items were removed. The original complete-scale results are retained as a robustness check in Appendix A.

Second, experimental manipulation checks and 2 × 2 between-group comparison analyses were conducted. Manipulation check items were first used to confirm whether participants effectively recognized the AI label and video quality manipulations; subsequently, 2 × 2 OLS regression models were employed to test the main effects and interaction effects of AI labels and video quality on perceived authenticity, trust, adoption intention, perceived risk, and related extended outcome variables, with simple effects analyses conducted where necessary.

Third, the core mechanism model was tested. Specifically, a basic trust model grounded in the HSM was first estimated to examine the effects of systematic and heuristic processing on trust; perceived authenticity and perceived risk were then incorporated to estimate a risk-extended trust model, testing the role of the risk pathway in trust formation; finally, the effects of trust and perceived risk on adoption intention were examined. Building on this, pathways related to sharing intention and the moderating role of user literacy were tested as supplementary analyses.

Fourth, mediation effects, moderation effects, and robustness were examined. Mediation effects were estimated using the bootstrap method to identify the indirect roles of systematic processing, trust, and perceived risk in the relevant pathways. The bootstrap method has been widely used for mediation testing because it does not require the assumption that indirect effects follow a normal distribution and provides more robust confidence interval estimates (Preacher & Hayes, 2008). Moderation analyses primarily examined whether AI literacy and media literacy alter the strength of the influence of AI labels on user trust and adoption intention. Additionally, this study employed the cSEM package in R (R version 4.5.2; cSEM version 0.6.1) to conduct supplementary composite model measurement invariance (MICOM) tests to assist in evaluating the comparability of key constructs across experimental groups. Given that this test primarily serves as a robustness assessment for cross-group interpretations, it is reported as a supplementary analysis in Appendix A rather than as core evidence in the main text.

## 4. Results

### 4.1. Measurement Model Evaluation and Manipulation Checks

Prior to formal hypothesis testing, this study first evaluated the measurement model. In response to the high empirical overlap between perceived authenticity and trust, the main analyses were conducted using refined item sets for these two constructs: perceived authenticity was measured using Auth1, Auth2, and Auth4, and trust was measured using Trust2 and Trust3. The original complete-scale results are retained as a robustness check in Appendix A. For most core constructs, Cronbach’s α and composite reliability (CR) exceeded 0.70, and the average variance extracted (AVE) was acceptable overall, indicating adequate internal consistency and convergent validity (see Table 4).

It should be noted that the AVE values for AI literacy and media literacy were relatively low, indicating that these competence-based variables exhibit considerable multidimensional characteristics and heterogeneity. Given that these two variables are primarily used as boundary condition variables rather than core mediating constructs in this study, they are employed mainly in moderation analyses, and the relevant results are interpreted with caution.

Regarding discriminant validity, the overall measurement model is reportable; however, an initial high empirical correlation existed between perceived authenticity and trust. To address this, we refined the scales by removing Auth3 (due to double-barreled loadings) and Trust1 (due to referent inconsistency). After refinement, the HTMT ratio decreased to 0.895, which provides improved and acceptable evidence of discriminant validity under the commonly used HTMT 0.90 criterion. The authenticity–trust relationship is still interpreted cautiously because the value remains close to the threshold. The original complete-scale results are retained as a robustness check in Appendix A, and the direction and significance of the core findings remain unchanged.

To further examine the stability of the scale structure, confirmatory factor analysis was conducted. The results showed satisfactory overall model fit, with CFI = 0.930, TLI = 0.919, RMSEA = 0.051, and standardized loadings for most items reaching acceptable levels. Additionally, exploratory factor analysis yielded KMO = 0.932 and a significant Bartlett’s test of sphericity (*p* < 0.001). The overall factor structure was largely consistent with the theoretical construct delineation (see Appendix A Table A3 for detailed supplementary measurement model results). Taken together, the measurement model demonstrates generally acceptable properties; however, the convergent validity of AI literacy and media literacy is relatively weak, and the empirical distinction between perceived authenticity and trust warrants continued caution in subsequent interpretations.

Regarding experimental manipulations, both the video quality and AI label manipulations achieved the expected effects. The video quality manipulation check showed that the high-quality group scored significantly higher than the low-quality group on “overall image and sound quality” (high-quality group: M = 5.547, SD = 1.091, *n* = 309; low-quality group: M = 4.552, SD = 1.421, *n* = 308; Welch t = 9.752, *p* < 0.001). The AI label recognition check was also significant, with a significant distributional difference between the labeled and unlabeled groups on the label recognition item (χ^2^ = 578.600, df = 2, *p* < 0.001). These results indicate that both experimental manipulations were effectively recognized by the participants, providing a foundation for subsequent hypothesis testing. Table 5 summarizes the manipulation check results.

### 4.2. Main Effects and Interaction Effects in the 2 × 2 Experiment

After confirming the validity of the manipulations, 2 × 2 OLS regression models were employed to examine the main effects and interaction effects of AI labels and video quality on key variables. The results show that the AI label had a significant negative main effect on perceived authenticity (coef = −0.214, *p* = 0.029), while the main effect of video quality on perceived authenticity was not significant (coef = 0.096, *p* = 0.329), and the interaction effect was also not significant (coef = −0.156, *p* = 0.262). These results indicate that AI-label condition was significantly negatively associated with users’ perceived authenticity of video content, supporting H1.

For trust, adoption intention, sharing intention, and perceived risk, neither the main effects of AI labels and video quality nor their interaction terms reached significance. Specifically, in the trust model, the main effect of video quality (coef = 0.080, *p* = 0.445), the main effect of the AI label (coef = −0.130, *p* = 0.216), and the interaction term (coef = −0.143, *p* = 0.334) were all non-significant; in the adoption intention model, the corresponding coefficients were 0.002 (*p* = 0.986), 0.091 (*p* = 0.463), and −0.088 (*p* = 0.613); in the sharing intention model, the corresponding coefficients were 0.123 (*p* = 0.395), −0.035 (*p* = 0.811), and −0.141 (*p* = 0.493); in the perceived risk model, the corresponding coefficients were −0.180 (*p* = 0.296), −0.173 (*p* = 0.314), and 0.227 (*p* = 0.350). Overall, the 2 × 2 experiment did not provide significant direct evidence that AI labels or video quality affected trust, adoption intention, sharing intention, or perceived risk. These findings suggest that the influence of external cues may operate less through simple direct effects and more through subsequent cognitive processing, trust formation, and risk assessment.

Regarding the exploratory research question (RQ1), supplementary 2 × 2 regression analyses further revealed a significant negative main effect of video quality on heuristic processing (coef = −0.310, *p* = 0.034), indicating that higher video quality was associated with a reduced tendency to rely on surface-level cues for judgment; however, neither the main effect of the AI label nor the interaction effect reached significance (see Appendix A Table A4). This result suggests that video quality, as a presentation signal, is more likely to exert its influence at the level of front-end cognitive processing rather than directly altering downstream behavioral intentions. At the same time, the existing evidence is insufficient to conclude that this effect would further translate stably into direct impacts on trust, adoption intention, or sharing intention. Table 6 reports the main effects and interaction effects in the 2 × 2 experiment.

### 4.3. Cognitive Processing Pathways, Perceived Risk, and Trust Formation

Having established that external cues did not exert significant simple direct effects on downstream outcomes, this study further examined the front-end cognitive processing pathways. The results showed that perceived authenticity was significantly negatively associated with cognitive load (coef = −0.607, *p* < 0.001), supporting H2; perceived authenticity was significantly positively associated with systematic processing (coef = 0.265, *p* < 0.001), supporting H3; cognitive load was significantly negatively associated with systematic processing (coef = −0.422, *p* < 0.001), supporting H4; cognitive load was significantly positively associated with heuristic processing (coef = 0.237, *p* < 0.001), supporting H5. In contrast, the direct association between perceived authenticity and heuristic processing was not significant (coef = −0.058, *p* = 0.215).

In the basic trust model, systematic processing was significantly positively associated with trust (coef = 0.478, *p* < 0.001), supporting H6; heuristic processing was significantly negatively associated with trust (coef = −0.112, *p* = 0.003), supporting H7. These results indicate that when encountering AI-generated video content with a certain comprehension threshold, systematic processing is associated with higher trust, while heuristic processing is associated with lower trust.

To further examine the risk pathway, this study first estimated the direct association between perceived authenticity and perceived risk. The results showed that perceived authenticity was significantly negatively associated with perceived risk (coef = −0.330, *p* < 0.001), supporting H8. A risk-extended trust model incorporating perceived authenticity, perceived risk, and systematic processing was then estimated. The results indicate that perceived authenticity was significantly positively associated with trust (coef = 0.535, *p* < 0.001), perceived risk was significantly negatively associated with trust (coef = −0.186, *p* < 0.001), and systematic processing also showed a significant positive association (coef = 0.206, *p* < 0.001), with a model R^2^ of 0.559. Furthermore, subsequent mediation analyses indicated that the indirect effect of the perceived authenticity → perceived risk → trust pathway was significant (indirect = 0.061, 95% CI [0.038, 0.079]), suggesting that the findings aligned with an indirect association between perceived authenticity and trust, involving a perceived risk pathway. These findings are consistent with the theoretical expectation of H9. Table 7 summarizes the cognitive processing pathways and trust formation results.

### 4.4. Trust, Perceived Risk, and Adoption Intention

At the proximal behavioral outcome level, this study focused on adoption intention. The core model incorporating systematic processing, heuristic processing, trust, and perceived risk demonstrated substantial explanatory power.

The adoption intention model (R^2^ = 0.469) showed the following results: trust was significantly positively associated with adoption intention (coef = 0.548, *p* < 0.001), while perceived risk was significantly negatively associated with adoption intention (coef = −0.152, *p* < 0.001). Trust and perceived risk were thus both significantly associated with adoption intention in the expected directions. Systematic processing retained a significant direct positive association with adoption intention (coef = 0.099, *p* = 0.007), whereas heuristic processing did not show a significant direct association (coef = 0.001, *p* = 0.970). Therefore, H10 and H11 are both supported. Table 8 reports the core regression results for trust, perceived risk, and adoption intention.

### 4.5. Extended Results and Boundary Condition Tests

To further assess the robustness of the core findings across extended outcome variables and boundary conditions, this study examined the main effects on sharing intention and related mediation pathways, as well as the moderating role of user literacy.

First, regarding the main effects on sharing intention, the sharing intention model (R^2^ = 0.502) indicated that neither systematic processing nor heuristic processing showed a significant direct association with sharing intention (*p* > 0.05); trust was significantly positively associated with sharing intention (coef = 0.617, *p* < 0.001), whereas perceived risk was significantly negatively associated with sharing intention (coef = −0.130, *p* < 0.001). The direction of the results for sharing intention is generally consistent with that of adoption intention, and H12 and H13 are supported.

Second, regarding the front-end cognitive mediation pathways, the results showed that the indirect effect of perceived authenticity → cognitive load → systematic processing was 0.239, 95% CI [0.176, 0.302]; the indirect effect of perceived authenticity → systematic processing → trust was 0.106, 95% CI [0.069, 0.151]. Furthermore, the indirect effect of perceived authenticity → perceived risk → trust was also significant (indirect = 0.061, 95% CI [0.039, 0.086]), further suggesting a statistically significant indirect association between perceived authenticity and trust involving a perceived-risk pathway.

Regarding the proximal dual-pathway mediation, the indirect effects of systematic processing → trust → adoption intention and systematic processing → trust → sharing intention were 0.261 and 0.294, respectively, both reaching significance. This aligns with a significant indirect association between systematic processing and higher behavioral intentions, consistent with a trust-mediated relationship. Combined with the earlier mediation results regarding the relationships among perceived authenticity, perceived risk, and trust, as well as the significant negative association between perceived risk and both adoption intention and sharing intention, these findings collectively provide supplementary evidence consistent with a proximal dual-pathway account centered on trust and perceived risk.

Regarding moderation effects, the interaction term label × AI literacy → trust was significant (coef = 0.160, *p* = 0.036), providing preliminary support for H14; the interaction term label × media literacy → adoption intention was also significant (coef = 0.168, *p* = 0.024), providing preliminary support for H15. The remaining interaction terms did not reach significance. This provides supplementary evidence that user literacy may constitute a boundary condition for the AI label effect, and suggests that this potential moderating role is primarily manifest in specific pathways.

**Figure 2:**
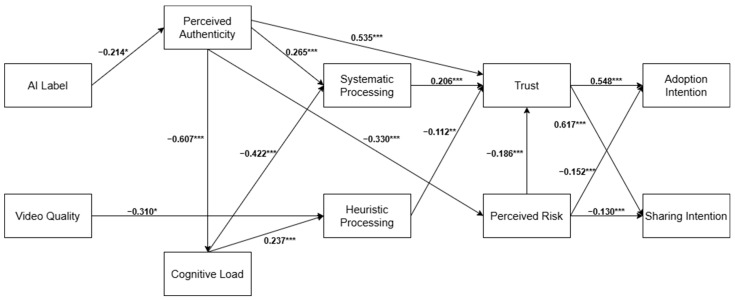
Summary of significant empirical findings. Solid arrows indicate statistically significant experimental effects or theory-guided associations in the tested direction. Coefficients are unstandardized regression coefficients drawn from the corresponding refined regression models reported in Section 4. Non-significant paths are omitted for clarity. Moderation effects are reported in Table 9 and are not displayed in this figure to avoid overcrowding. * *p* < 0.05, ** *p* < 0.01, *** *p* < 0.001. Mechanism pathways among self-reported variables should be interpreted as associations rather than strictly causal effects.

To facilitate a clearer visual interpretation of the empirical findings across the structural model, the main statistically significant experimental effects and theory-guided associations are summarized in Figure 2. Non-significant pathways are omitted for visual clarity, and the significant moderation effects are reported in Table 9 rather than displayed in the figure to avoid overcrowding.

### 4.6. Robustness Checks

To further examine the robustness of the study’s conclusions, measurement model test results and MICOM test results are reported in Appendix A. Overall, the findings regarding the associations among the core constructs remained consistent, specifically the negative relationship between AI labels and perceived authenticity, the positive association between systematic processing and trust, and the predictive value of trust and perceived risk for behavioral intentions, indicating that the core conclusions possess reasonable stability. It should be noted that the original complete-scale version exhibited high construct correlations, particularly an initial HTMT value of 0.937 between perceived authenticity and trust. Nevertheless, when the analyses were re-estimated using the original complete-scale scores as a robustness check, the direction and significance of the core findings remained unchanged.

To assist in evaluating the comparability of key constructs across groups, this study further employed the cSEM package in R to conduct composite model measurement invariance (MICOM) tests based on 5000 permutation samples. The results showed that in the AI label grouping (label absent vs. label present), the composite invariance tests (Step 2) for all 11 constructs were non-significant (*p* > 0.05), indicating that all achieved partial measurement invariance; however, a few constructs, including perceived authenticity, trust, and perceived risk, did not simultaneously satisfy mean and variance equality (Step 3), and thus did not achieve full measurement invariance. In the video quality grouping (high vs. low), all 10 constructs except for the perceived quality manipulation-check variable passed the composite invariance test; the perceived quality variable did not establish composite invariance (*p* = 0.012). This is closely related to the fact that video quality itself serves as an experimental manipulation dimension, and it also suggests that cross-group interpretations involving this variable should be approached with caution. Overall, the MICOM tests indicate that between-group comparisons for most core constructs have a reasonable basis for comparability; however, caution remains warranted for cross-group inferences involving the perceived quality manipulation-check variable and a few specific constructs. Detailed results are presented in Appendix A Table A5 and Table A6.

## 5. Discussion

This study addressed the question of how AI labels and video quality influence user responses to AI-generated video content by constructing and testing an integrated model of “external cues—cognitive processing—trust/risk—behavioral intention”. Based on a 2 × 2 between-subjects experiment and multi-level mechanism analysis, the results indicate that AI labels significantly reduce users’ perceived authenticity of video content but do not directly alter their trust, adoption intention, or sharing intention. Users’ ultimate responses to AI-generated video content were not directly altered by external labeling cues alone but appeared to be more closely associated with front-end cognitive processing and subsequent trust–risk evaluations.

### 5.1. Discussion of Findings

The most direct finding of this study is that the AI-label condition was significantly negatively associated with users’ perceived authenticity of video content but did not directly increase or decrease trust, adoption intention, or sharing intention. This suggests that in the context of AI-generated video content, transparent disclosure does not automatically translate into higher trust. Users do not naturally increase their acceptance simply because a platform or content creator explicitly discloses that the content is AI-generated; rather, AI labels appear more likely to first trigger a cautious evaluation of the content’s authenticity and reliability.

This result carries two implications. First, the effect of AI labels operates primarily at the initial judgment stage rather than directly influencing ultimate behavioral intentions. This is consistent with the argument presented earlier in this paper, namely that transparency labels are more likely to prompt users to further scrutinize the source and reliability of content than to serve straightforwardly as a positive signal. Second, the 2 × 2 experimental results showed that video quality did not significantly alter this labeling effect; accordingly, this study did not observe significant direct evidence that labeling alone enhances trust or alleviates users’ concerns. This implies that in content contexts with a certain cognitive threshold, such as AI-generated video content, users do not make overall acceptance judgments based solely on whether disclosure occurs; rather, the label primarily opens an entry point for subsequent cognitive processing.

The findings also indicate that the heuristic–systematic model retains explanatory relevance in the context of AI-generated video content. Perceived authenticity was significantly negatively associated with cognitive load and was associated with greater systematic processing, while cognitive load was significantly negatively associated with systematic processing and positively associated with heuristic processing. This suggests that when users encounter AI-generated video content, their responses do not stop at the label itself but are more likely linked to a set of front-end cognitive patterns comprising authenticity judgments, cognitive load, and processing modes. In other words, external labeling cues do not directly correspond to final acceptance or rejection but appear to first influence users’ initial content judgments and their subsequent processing tendencies.

Furthermore, systematic processing had a significant positive association with trust, while heuristic processing had a significant negative association with trust. This indicates that for video content involving relatively high comprehension demands, higher levels of systematic processing typically accompany higher trust, whereas stronger heuristic processing tends to coexist with lower trust. It should be emphasized that given that the relevant variables primarily derive from self-report measures collected at a single time point, this finding is best understood as an association pattern consistent with theoretical expectations rather than as strict identification of sequential psychological processes. Nevertheless, this result suggests that in the context of AI-generated video content, users who more carefully evaluate content logic, evidence quality, and explanatory processes tend to form more stable trust judgments; conversely, those who rely more heavily on superficial cues and quick impressions find it more difficult to establish robust trust.

Another core finding is that the overall pattern is consistent with a proximal dual-pathway account centered on trust and perceived risk. Supplementary analyses further indicate that the direction of results for sharing intention is generally consistent with that for adoption intention. Trust was significantly positively associated with both adoption intention and sharing intention, while perceived risk was significantly negatively associated with both. Additionally, in the extended trust model, perceived authenticity was significantly associated with higher trust, and perceived risk was significantly associated with lower trust, suggesting that users’ trust judgments regarding AI-generated video content do not arise in isolation but are more likely connected to authenticity assessments, information processing, and risk evaluations. Notably, mediation tests further revealed that systematic processing was significantly and indirectly associated with higher trust and higher behavioral intentions, and the results were consistent with an indirect association between perceived authenticity and trust through perceived risk. Taken together, the findings are broadly consistent with a “trust promotion–risk inhibition” dual-pathway framework rather than a single-pathway account.

This study’s simultaneous examination of both adoption intention and sharing intention has also proven informative. Although both dependent variables were significantly associated with trust and perceived risk, their proximal structures were not entirely identical: systematic processing retained a significant direct association with adoption intention, whereas its direct association with sharing intention was not significant. This suggests that compared with “whether I am willing to continue using this type of content”, “whether I am willing to recommend it to others” may better reflect responsibility judgments and social choices regarding AI-generated video content in real-world dissemination contexts. Evaluation of AI content cannot be limited solely to the individual’s own usage level. Particularly in digital environments, when a user recommends a specific piece of AI-generated video content, this often implies not only a judgment about the content itself, but also an implicit assessment of content quality, information accuracy, and the consequences of others’ acceptance.

Finally, this study found that AI literacy moderates the association between AI labels and trust, while media literacy moderates the association between AI labels and adoption intention. This suggests that AI label effects are not entirely uniform across users. However, given the relatively weak convergent validity of the AI literacy and media literacy measures, these results are better regarded as supplementary boundary evidence rather than core conclusions. Their more important implication is that the effectiveness of external labeling cues depends not only on the label itself, but also on whether users possess the capacity to understand the label, interpret its meaning, and contextualize it within specific content.

It should also be noted that although the refined item sets improved discriminant validity, the HTMT value between perceived authenticity and trust remained at 0.895—close to the commonly used 0.90 threshold. This suggests that in the context of AI-generated video content, the two constructs remain highly related empirically, and some respondents may form relatively similar overall judgments regarding “whether the content is authentic and reliable” and “whether I trust this video”. It should also be noted that although the refined item sets improved discriminant validity, the HTMT value between perceived authenticity and trust remains at 0.895—close to the commonly used 0.90 threshold. This suggests that in the context of AI-generated video content, the two constructs remain highly related empirically, and some respondents may form relatively similar overall judgments regarding “whether the content is authentic and reliable” and “whether I trust this video”. Therefore, the perceived authenticity–trust pathway reported in this study should not be interpreted as reflecting two fully independent psychological processes. While the theoretical distinction between the two constructs is maintained, the empirical findings are interpreted with caution, and claims about the independence of these constructs are deliberately conservative.

### 5.2. Theoretical Contributions

The theoretical contributions of this study are primarily reflected in three aspects.

First, this study extends the heuristic–systematic model to the context of AI-generated video content, which combines content-evaluative and media-related characteristics. Existing HSM research has primarily focused on general information processing and persuasion contexts; this study further demonstrates that in AI-generated video content requiring substantial cognitive engagement, users’ responses to content can still be understood through the distinction between systematic and heuristic processing. In particular, video quality as a presentation signal primarily serves to reduce the tendency toward front-end heuristic processing rather than directly altering downstream adoption attitudes, providing new contextual evidence for the application of the HSM in generative AI content settings.

Second, building upon the HSM, this study incorporates trust and perceived risk to propose and test a “trust promotion–risk inhibition” dual-pathway account. Compared with research perspectives that treat trust solely as a single antecedent of behavioral intention, this study indicates that users’ acceptance of AI-generated video content is more likely associated with both trust enhancement and risk reduction as two types of proximal evaluations. Supplementary analyses further show that this framework also exhibits a generally consistent direction for sharing intention. This finding contributes to a more complete understanding that acceptance of AI content is not a single attitudinal outcome but rather a behavioral tendency shaped by the combined influence of multiple evaluations.

Third, this study extends AI acceptance research from general tool adoption to the specific context of AI-generated video content, which involves content consumption and dissemination. Unlike general AI tools, AI-generated video content involves not only the decision of whether to use a technology but also dimensions such as content authenticity, information comprehension, dissemination responsibility, and the potential influence on others. The results suggest that users’ responses to AI-generated video content resemble a continuous evaluation process in which content judgments, processing modes, trust, and risk are interrelated. This provides a more content-specific research perspective for understanding the acceptance of generative AI content in digital environments.

Finally, it should be noted that although this study incorporates perceived authenticity and trust into the same explanatory framework, the two are highly correlated at the empirical level. This study therefore interprets them as two judgment dimensions that are theoretically distinguishable but closely linked in actual evaluations, rather than as independent psychological stages with entirely clear boundaries.

### 5.3. Practical Implications

The findings of this study offer direct implications for digital platforms, AI content producers, and platform governance bodies.

First, simply labeling content as “AI-generated” is insufficient to enhance user acceptance. The primary direct effect of such labeling is to reduce perceived authenticity rather than to automatically generate trust. Consequently, if platforms aim to promote broader acceptance of AI-generated video content, transparent disclosure alone is insufficient; it must be complemented by more comprehensive content descriptions, source explanations, and quality assurance mechanisms.

Second, the key to enhancing user acceptance and recommendation lies in building trust and reducing perceived risk. In practice, this means that platforms and content creators should attend more closely to the logical coherence, professionalism, and verifiability of content, as trust judgments in online information environments are influenced not only by source information, but also by the combined effects of content organization and presentation design (König et al., 2019). For example, providing clearer source explanations, human-review indicators, expert-validated information, or basic descriptions of the generation process may help alleviate users’ concerns about misinformation and unclear accountability.

Third, while video quality does not directly enhance acceptance or recommendation, it does influence front-end processing. This suggests that when designing AI-generated video content, platforms should still attend to audiovisual presentation quality, as higher-quality visuals and audio can significantly reduce users’ reliance on intuitive or superficial cues for hasty judgment—that is, reduce heuristic processing tendencies—thereby providing more stable front-end cognitive conditions for objective content evaluation.

Finally, user literacy development is equally important. Given that AI literacy and media literacy appear to alter how AI labels function, platform governance should focus not only on “how content is labeled” but also on “how users interpret these labels”. In the long run, enhancing users’ understanding of the boundaries of AI technology, content sources, and media information judgment may prove more effective for governance than simply increasing the number of labels.

### 5.4. Limitations and Future Directions

Although this study yielded useful findings, several limitations should be acknowledged.

First, the sample in this study predominantly consisted of young, highly educated students who reported frequent use of generative AI. While this demographic represents a primary audience for digital AI content, their familiarity with the technology may make them less sensitive to AI transparency labels than the general public. Future research should recruit more diverse and representative samples to validate whether the current findings hold across different age groups and tech-literacy levels. Second, this study used an explanatory video about the “Pomodoro Technique” as the sole stimulus material. Because this topic is relatively neutral and low-risk, it may not trigger strong concerns about misinformation or personal harm. Users might react very differently to AI-generated content in high-stakes or sensitive domains, such as news, healthcare, or finance, where AI labels might more directly activate risk perceptions and reduce trust. Future research should incorporate a wider variety of video materials to test the stability of the proposed model across varying levels of topic sensitivity and content complexity.

Third, this study deliberately restricted the definition of “video quality” to audiovisual presentation quality to isolate it as an observable presentation signal, holding informational content constant across conditions. Consequently, this study did not measure factual accuracy or informational truthfulness. In reality, high visual quality might act as a credibility heuristic that masks false information—a critical risk in AI-generated media. Future research must decompose the concept of “quality” at a finer level to examine how visual appeal and informational accuracy interact in shaping trust and risk perceptions.

Fourth, although the supplementary model comparison results indicate that a two-factor model distinguishing perceived authenticity and trust yields better overall fit, a high empirical correlation persists between the two. Future research should develop measurement tools better tailored to generative content contexts to more precisely capture the transition from content evaluation to overall trust judgment. Additionally, the convergent validity of the AI literacy and media literacy scales is relatively weak; therefore, the findings regarding boundary conditions should be regarded as preliminary evidence. Future research could develop literacy scales more suited to generative AI content contexts to further test the stability of the relevant moderating relationships.

Fifth, this study employed an online situational experiment focused on assessing users’ immediate reactions to a single, first-time exposure to AI-generated video content, treating AI labels and video quality as static external cues. This design may oversimplify the complex, iterative ways users interact with AI-generated media over time. It is particularly important to note that while this study employed rigorous between-subjects experimental manipulation of AI labels and video quality, the mediating and dependent variables—including cognitive processing, trust, perceived risk, and behavioral intentions—were all measured via self-report at a single time point. Consequently, the mediation pathways presented in this study primarily reflect statistically significant associations supported by theory rather than strictly sequential causal identification. Future research could incorporate longitudinal designs, process-tracing methods, or cognitive neuroscience evidence to further examine this cognition–trust mechanism.

Furthermore, supplementary MICOM tests indicate that the perceived quality manipulation-check variable did not establish compositional invariance between the high- and low-quality groups, and some core constructs did not achieve full measurement invariance in between-group comparisons. Therefore, caution should be exercised when making rigorous cross-group inferences involving these constructs in future research.

Finally, it should be noted that this study focused on user responses to AI-generated content rather than objective learning or performance outcomes. The experimental materials were used as a controlled content context rather than as an educational intervention. Future research could further examine how these cognitive and trust mechanisms operate in settings involving formal learning tasks or knowledge acquisition outcomes.

## 6. Conclusions

This study focused on AI-generated video content and constructed and tested an integrated framework of “external cues—cognitive processing—trust/risk—behavioral intention” to examine how AI labels and video quality influence user responses. The findings reveal that AI-label condition was significantly negatively associated with users’ perceived authenticity of video content but did not directly alter their trust or adoption intention; simultaneously, video quality as a presentation signal was significantly associated with reduced heuristic processing tendencies. In subsequent models, systematic processing was significantly associated with higher trust, while heuristic processing was significantly associated with lower trust; trust was significantly positively associated with adoption intention, whereas perceived risk was significantly negatively associated with it. Further analyses indicate that the direction of results for sharing intention was generally consistent with that for adoption intention, while AI literacy and media literacy provided supplementary evidence for potential boundary differences in the AI label effect.

Overall, this study suggests that in the context of AI-generated video content, users’ ultimate responses are not directly determined by external labeling cues. Rather than addressing the simple question of whether transparent disclosure is effective, the findings lend support to an explanatory framework that integrates front-end content evaluation, cognitive processing, trust, and risk assessment. For generative AI content governance, while transparent disclosure remains necessary, users’ attitudes toward acceptance and dissemination appear more likely to depend on their comprehensive assessment of content authenticity, credibility, and potential risks. However, it is important to note that these conclusions were drawn from a sample consisting predominantly of young (mean age ≈ 22 years), highly educated, and digitally experienced users who evaluated a specific low-risk explanatory video regarding the Pomodoro Technique. The findings should therefore be interpreted as applying primarily to this population and content context, rather than to AI-generated video content or users in general. Further research is necessary to confirm whether these cognitive and trust dynamics hold across more diverse demographic groups, higher-stakes information domains (e.g., news, health, finance), and a broader range of AI-generated content types.

## Figures and Tables

**Table 1:** Definitions of key constructs and core literature sources.

Construct	Definition	Core Literature Sources
Perceived authenticity	An individual’s subjective perception of the authenticity, naturalness, and credibility of AI-generated video content	F. Li and Yang (2024)
Cognitive load	The degree of cognitive effort perceived by an individual during the process of understanding, evaluating, and processing video information	S. Chen and Chaiken (1999); Petty and Cacioppo (1986)
Systematic processing	A processing mode in which individuals engage in in-depth analysis, evidence integration, and logical judgment of video information	Chaiken (1980); S. Chen and Chaiken (1999)
Heuristic processing	A processing mode in which individuals rely on surface-level cues, intuitive impressions, and simplified rules to form judgments	Chaiken (1980); S. Chen and Chaiken (1999)
Trust	An individual’s overall assessment of the reliability and dependability of AI-generated video content and their generation mechanisms	F. Li and Yang (2024); Y. Li et al. (2025)
Perceived risk	The degree to which individuals are concerned that AI-generated video content may lead to misinformation, inaccuracies, or negative consequences	Featherman and Pavlou (2003)
Adoption intention	An individual’s behavioral tendency to continue adopting or using AI-generated video content	Venkatesh et al. (2003)
Sharing intention	An individual’s behavioral tendency to recommend AI-generated video content to others	F. Li and Yang (2024)
AI literacy	An individual’s level of knowledge and ability regarding the principles, capabilities, and limitations of AI	Ng et al. (2021); Long and Magerko (2020)
Media literacy	An individual’s ability to identify, analyze, and evaluate media content	Anstead et al. (2025); Peng et al. (2024)

**Table 2:** Demographic characteristics of the sample (*N* = 617).

Variable	Category	*N*	Percentage (%)
Gender	Male	197	31.9
	Female	420	68.1
Age	Mean (SD)	21.76 (1.75)	—
Education	Currently enrolled in undergraduate program	356	57.7
	Currently pursuing a master’s degree	123	19.9
	Bachelor’s degree	120	19.4
	Master’s degree or higher	9	1.5
	High school or below	9	1.5
Frequency of watching video content	Often/Almost every day	259	42.0
	Sometimes/Occasionally/Rarely	358	58.0
Frequency of generative AI use	Often/Almost every day	512	83.0
	Sometimes/Occasionally	105	17.0

**Table 3:** Sample distribution and experimental conditions (*N* = 617).

Experimental Condition	Sample Size	AI Label	Video Quality
Low quality + No label	154	Absent	Low
Low quality + AI label	154	Present	Low
High quality + No label	154	Absent	High
High quality + AI label	155	Present	High
Total	617		

**Table 4:** Measurement and validity results for key variables (*N* = 617).

Construct	Number of Items	Cronbach’s α	CR	AVE
Perceived authenticity	3	0.809	0.810	0.587
Cognitive load	4	0.800	0.805	0.510
Systematic processing	4	0.855	0.856	0.598
Heuristic processing	4	0.831	0.833	0.564
Trust	2	0.756	0.758	0.611
Adoption intention	3	0.843	0.848	0.652
Sharing intention	3	0.892	0.894	0.738
Perceived risk	3	0.895	0.897	0.745
AI literacy	4	0.768	0.772	0.467
Media literacy	4	0.685	0.697	0.367

**Table 5:** Manipulation check results.

Test Item	Group/Statistic	Value	*p*-Value
Video quality manipulation	High-quality group mean (SD)	5.547 (1.091)	—
	Low-quality group mean (SD)	4.552 (1.421)	—
	Welch t	9.752 ***	<0.001
AI label manipulation	Chi-square χ^2^	578.600 ***	<0.001

Note: *** *p* < 0.001.

**Table 6:** Main effects and interaction effects in the 2 × 2 experiment (*N* = 617).

Dependent Variable	Predictor	Coefficient	*p*-Value
Perceived authenticity	Video quality	0.096	0.329
	AI label	−0.214	0.029 *
	Video quality × AI label	−0.156	0.262
Trust	Video quality	0.080	0.445
	AI label	−0.130	0.216
	Video quality × AI label	−0.143	0.334
Adoption intention	Video quality	0.002	0.986
	AI label	0.091	0.463
	Video quality × AI label	−0.088	0.613
Sharing intention	Video quality	0.123	0.395
	AI label	−0.035	0.811
	Video quality × AI label	−0.141	0.493
Perceived risk	Video quality	−0.180	0.296
	AI label	−0.173	0.314
	Video quality × AI label	0.227	0.350

Note: * *p* < 0.05.

**Table 7:** Cognitive processing pathways and trust formation results.

Path	Coefficient	*p*-Value	Result
Perceived authenticity → Cognitive load	−0.607	<0.001	Supports H2
Perceived authenticity → Systematic processing	0.265	<0.001	Supports H3
Cognitive load → Systematic processing	−0.422	<0.001	Supports H4
Cognitive load → Heuristic processing	0.237	<0.001	Supports H5
Perceived authenticity → Heuristic processing	−0.058	0.215	Not supported
Perceived authenticity → Perceived risk	−0.330	<0.001	Supports H8
Systematic processing → Trust	0.478	<0.001	Supports H6
Heuristic processing → Trust	−0.112	0.003	Supports H7
Perceived authenticity → Trust (extended model)	0.535	<0.001	—
Perceived risk → Trust (extended model)	−0.186	<0.001	Supports H9
Systematic processing → Trust (extended model)	0.206	<0.001	—

Note: The first five paths correspond to the front-end cognitive processing model; the sixth path corresponds to the perceived risk direct effect model; the seventh and eighth paths are based on the basic HSM trust model; the remaining paths are based on the risk-extended model.

**Table 8:** Core regression results for trust, perceived risk, and adoption intention.

Dependent Variable	Independent Variable	Coefficient	*p*-Value	R^2^
Adoption intention	Systematic processing	0.099	0.007 **	0.469
	Heuristic processing	0.001	0.970	
	Trust	0.548	<0.001 ***	
	Perceived risk	−0.152	<0.001 ***	

Note: ** *p* < 0.01, *** *p* < 0.001.

**Table 9:** Key mediation and moderation effect results.

Type	Path	Indirect Effect/Coefficient	95% CI/*p*-Value	Result
Mediation	Perceived authenticity → Cognitive load → Systematic processing	0.239	[0.176, 0.302]	Significant
Mediation	Perceived authenticity → Systematic processing → Trust	0.106	[0.069, 0.151]	Significant
Mediation	Perceived authenticity → Perceived risk → Trust	0.061	[0.039, 0.086]	Significant
Mediation	Systematic processing → Trust → Adoption intention	0.261	[0.203, 0.321]	Significant
Mediation	Systematic processing → Trust → Sharing intention	0.294	[0.232, 0.360]	Significant
Moderation	Label × AI Literacy → Trust	0.160	*p* = 0.036 *	Preliminary support for H14
Moderation	Label × Media Literacy → Adoption intention	0.168	*p* = 0.024 *	Preliminary support for H15

Note: * *p* < 0.05. Mediation effects are based on bootstrap resampling; significance is indicated when the confidence interval does not include 0. Moderation results are based on mean-centered variables.

## Data Availability

The datasets generated during and/or analyzed during the current study are available from the corresponding author on reasonable request.

## Appendix A

**Table A1:** Key construct items, item numbers, and literature sources.

Construct	Item Number	Primary Literature Source	Description
Systematic processing	Q5–Q8	Chaiken (1980); S. Chen and Chaiken (1999)	Adapted from classic HSM scales for the AI-generated video content context
Heuristic processing	Q9–Q12	Chaiken (1980); S. Chen and Chaiken (1999)	Adapted from classic HSM scales for the AI-generated video content context
Perceived authenticity	Q13–Q16	F. Li and Yang (2024)	Adapted from AIGC label research with contextual revisions
Trust	Q17–Q19	F. Li and Yang (2024)	Adapted from the AIGC content trust scale with contextual revisions
Perceived quality	Q20–Q22	Xu and Sundar (2016)	Used as a manipulation check for the video quality manipulation
Sharing intention	Q23–Q25	F. Li and Yang (2024)	Adapted from the AIGC sharing/recommendation intention scale
Adoption intention	Q26–Q28	Venkatesh et al. (2003)	Adapted from the UTAUT behavioral intention construct
Perceived risk	Q29–Q31	Featherman and Pavlou (2003); revised for AI content context	Focuses on information accuracy risk, learning consequence risk, and general negative consequence risk
Cognitive load	Q32–Q35	S. Chen and Chaiken (1999); Petty and Cacioppo (1986)	Reflects ease of understanding, evaluation capacity, and processing burden
AI literacy	Q36–Q39	Ng et al. (2021); Long and Magerko (2020)	Reflects understanding of AI principles, recognition ability, and technical awareness
Media literacy	Q40–Q43	Ashley et al. (2013); Livingstone (2004)	Reflects source verification, bias recognition, and misinformation detection

Note: All original items were collected using a seven-point Likert scale (1 = strongly disagree, 7 = strongly agree). In the main analysis, Auth3 and Trust1 were removed to improve discriminant validity between perceived authenticity and trust. The original complete-scale results were retained as a robustness check.

**Table A2:** Randomization check results for experimental groups.

Variable	Test Method	Statistic	*p*-Value
Gender	Chi-square test	χ^2^(3) = 0.919	0.821
Education	Chi-square test	χ^2^(12) = 7.946	0.789
Age	One-way ANOVA	F(3, 613) = 0.842	0.472
Frequency of watching video content	Chi-square test	χ^2^(12) = 3.632	0.989
Frequency of generative AI use	Chi-square test	χ^2^(9) = 6.702	0.668

**Table A3:** Supplementary measurement model results.

Indicator	Value
KMO	0.932
Bartlett’s test of sphericity	*p* < 0.001
CFA: CFI	0.930
CFA: TLI	0.919
CFA: RMSEA	0.051
Maximum HTMT (Original scale)	0.937
Maximum HTMT (refined scale)	0.895

Note: CFA = confirmatory factor analysis; EFA = exploratory factor analysis. The original complete-scale model produced a maximum HTMT value of 0.937 between perceived authenticity and trust. After removing Auth3 and Trust1 as described in Section 4.1, the maximum HTMT value decreased to 0.895.

**Table A4:** The 2 × 2 regression results for front-end processing variables.

Dependent Variable	Predictor	Coefficient	*p*-Value
Heuristic processing	Video quality	−0.310	0.034 *
	AI label	0.091	0.537
	Video quality × AI label	0.150	0.369
Systematic processing	Video quality	0.151	0.297
	AI label	−0.090	0.537
	Video quality × AI label	−0.134	0.410
Cognitive load	Video quality	−0.227	0.108
	AI label	0.114	0.422
	Video quality × AI label	0.168	0.289

Note: * *p* < 0.05. This table reports the 2 × 2 regression results for front-end processing variables.

**Table A5:** MICOM results for AI label groups (label absent vs. label present).

Construct	Step 2 Composite Invariance (c)	Permutation *p*-Value	Step 3 Mean Equality (*p*)	Step 3 Variance Equality (*p*)	Conclusion
Perceived authenticity	0.9893	0.3704	0.0002 ***	0.0404 *	Partial measurement invariance
Cognitive load	0.9426	0.1342	0.6014	0.8126	Full measurement invariance
AI literacy	0.8956	0.2800	0.4046	0.2416	Full measurement invariance
Media literacy	0.8587	0.0556	0.4956	0.5998	Full measurement invariance
Perceived quality	0.8390	0.2256	0.9364	0.3982	Full measurement invariance
Perceived risk	0.9906	0.6878	0.8738	0.0338 *	Partial measurement invariance
Systematic processing	0.9953	0.8664	0.5404	0.1284	Full measurement invariance
Heuristic processing	0.9451	0.4922	0.3582	0.1400	Full measurement invariance
Trust	0.9975	0.4918	0.0056 **	0.0530	Partial measurement invariance
Adoption intention	0.9909	0.3700	0.4752	0.5710	Full measurement invariance
Sharing intention	0.9856	0.1758	0.2330	0.4226	Full measurement invariance

Note: Based on 5000 permutation samples. * *p* < 0.05, ** *p* < 0.01, *** *p* < 0.001. Sample sizes: label absent = 308, label present = 309.

**Table A6:** MICOM results for video quality groups (high quality vs. low quality).

Construct	Step 2 Composite Invariance (c)	Permutation *p*-Value	Step 3 Mean Equality (*p*)	Step 3 Variance Equality (*p*)	Conclusion
Perceived authenticity	0.9853	0.2408	0.8158	0.2582	Full measurement invariance
Cognitive load	0.9338	0.0832	0.5032	0.4114	Full measurement invariance
AI literacy	0.9039	0.3210	0.7978	0.4830	Full measurement invariance
Media literacy	0.8917	0.1160	0.3828	0.8714	Full measurement invariance
Perceived quality	0.6054	0.0124 *	0.0216 *	0.9764	Compositional invariance not established
Perceived risk	0.9762	0.3888	0.6074	0.5928	Full measurement invariance
Systematic processing	0.9917	0.7248	0.7078	0.7322	Full measurement invariance
Heuristic processing	0.9698	0.7412	0.1676	0.1630	Full measurement invariance
Trust	0.9998	0.9526	0.8938	0.7034	Full measurement invariance
Adoption intention	0.9794	0.1074	0.6448	0.2290	Full measurement invariance
Sharing intention	0.9923	0.4044	0.6474	0.7176	Full measurement invariance

Note: Based on 5000 permutation samples. * *p* < 0.05. Sample sizes: high quality = 309, low quality = 308.
